# No strong association among epigenetic modifications by DNA methylation, telomere length, and physical fitness in biological aging

**DOI:** 10.1007/s10522-022-10011-0

**Published:** 2023-01-02

**Authors:** Yasuhiro Seki, Dora Aczel, Ferenc Torma, Matyas Jokai, Anita Boros, Katsuhiko Suzuki, Mitsuru Higuchi, Kumpei Tanisawa, Istvan Boldogh, Steve Horvath, Zsolt Radak

**Affiliations:** 1 Faculty of Sport Sciences, Waseda University, Tokorozawa, 2-579-15 Japan; 2 Research Institute of Sport Science, Hungarian University of Sport Science, Budapest, Hungary; 3 Department of Microbiology and Immunology, University of Texas Medical Branch at Galveston, Galveston, TX 77555 USA; 4 Department of Human Genetics, David Geffen School of Medicine, University of California Los Angeles, Los Angeles, CA 90095 USA

**Keywords:** Telomere, Exercise, Physical fitness, DNA methylation, Epigenetical aging

## Abstract

Cellular senescence is greatly accelerated by telomere shortening, and the steps forward in human aging are strongly influenced by environmental and lifestyle factors, whether DNA methylation (DNAm) is affected by exercise training, remains unclear. In the present study, we investigated the relationships between physiological functions, maximal oxygen uptake (VO2max), vertical jump, working memory, telomere length (TL) assessed by RT-PCR, DNA methylation-based estimation of TL (DNAmTL), and DNA methylation-based biomarkers of aging of master rowers (N = 146) and sedentary subjects (N = 95), aged between 37 and 85 years. It was found that the TL inversely correlated with chronological age. We could not detect an association between telomere length and VO2max, vertical jump, and working memory by RT-PCR method, while these physiological test results showed a correlation with DNAmTL. DNAmGrimAge and DNAmPhenoAge acceleration were inversely associated with telomere length assessed by both methods. It appears that there are no strong beneficial effects of exercise or physiological fitness on telomere shortening, however, the degree of DNA methylation is associated with telomere length.

## Introduction

Healthy aging with relatively high physiological function is the goal of human society. To achieve this, it would be important to understand the aging process and then develop interventions that help to reach the goals. One of the mechanisms, known to regulate the aging process is based on the limiting factors of cell proliferation, namely on telomeres (Greider 1996). Telomeres are protective caps on the ends of chromosomes with repeated deoxyribonucleic acid sequences rich in TTAGGG, between 3- and 20 kb- long in humans (Hande 2004). Telomeres are complexes comprising not only (T2AG3)n DNA repeats, but also protecting loops, including proteins and RNA. Attrition disturbs these loops, which triggers DNA damage response and then cellular senescence and inflammation (Greider 1996). It also has to be mentioned, that importance of the telomere to evaluate the progress of aging is under debate (Simons 2015).

Oxidative stress causes accelerated telomere shortening primarily through the oxidation of guanine due to its lowest oxidation potential among nucleic acid bases, especially in telomere quadruplexes (Radak and Boldogh 2010). After a certain number of cell divisions, which results in loss of telomere length, dividing cells cannot replicate anymore reaching cellular senescent (Linskens et al. 1995). Although the enzyme of telomerase can add de novo base pairs to telomere (Greider and Blackburn 1989), the age-related shortening is well documented (Campisi 2005; Bize et al. 2009). Indeed, accumulating evidence suggests a strong link between telomere length and aging and age-associated diseases (Morin 1997; Sikora 2013; Fyhrquist and Saijonmaa 2012; Sahin and DePinho 2012).

It has been shown that telomere length (TL) is dependent on various lifestyle factors. It has been shown that in preschool children obesity was linked to reduced levels of docosahexaenoic acid, an increased arachidonic acid/ docosahexaenoic acid ratio, and shortened telomere in leukocytes (Liu et al. 2021). Short-term administration of nicotinamide mononucleotide resulted in significant elongation of TL in peripheral blood mononuclear cells of C57BL/6 mice (Niu et al. 2021). Studies suggest that physical exercise also has beneficial effects on TL (Lee et al. 2013; Manoy et al. 2020; Loprinzi et al 2015). Even a short-term exercise program for 12 weeks, with low frequency, moderate intensity, and explosive-type resistance training could have beneficial effects on telomeres. Indeed, above mentioned explosive resistance training lessened telomere shortening and correlated with the amelioration of redox homeostasis (Dimauro et al. 2016).

Because aging is strongly influenced by lifestyle Horvath and Hannum developed DNA methylation-based epigenetic aging clocks, which more precisely reflect aging than chronological age (Horvath 2015; Hannum et al. 2013). Moreover, in a huge cohort (n = 5713) it has been shown that there is an interaction between genome-wide methylation, TL, and epigenetic aging (Lee et al. 2019). Based on previous studies, we hypothesize that the level of physical fitness would affect epigenetic aging clocks and TL of whole blood samples in aged individuals. Moreover, we aimed to compare the interactions between physiological test results and TL assessed by the RT-PCR method and DNA methylation-based estimation (Pearce et al. 2022).

## Methods

### Subjects

Subjects were volunteers, who participated in the 2019 Masters World Rowing Championships in Venice, Hungary, and aged-matched sedentary individuals. The master rowers were recruited at the championships using pamphlets, while sedentary subjects were by calls published in a newspaper in Budapest. The investigation was carried out voluntarily with the ethics license provided by the Hungarian Scientific and Research Ethical Committee 25167-6/2019/EUIG. Our cohort consisted of a total of 241 people, 146 masters (mean age 59 ± 9.7 years) and 95 sedentary (mean age 62 ± 12.3 years) (Table 1).

**Table 1:** Characteristics and results of the subjects

n = 241	Master rowers	Sedentary subjects
Number of patients	146	94
Age (years), Mean ± SD	59 ± 9.7	62 ± 12.3
Male	78	30
Age (years), Mean ± SD	60.0 ± 10.6	60.5 ± 14
Female	68	65
Age (years), Mean ± SD	57.1 ± 8.4	63.4 ± 11.4
TL, Mean ± SD	8.57 ± 0.36	8.56 ± 0.34
DNAmTL, Mean ± SD	6.85 ± 0.27	6.79 ± 0.29
BMI (kg/m^2^), Mean ± SD	24.48 ± 2.8	27.01 ± 4.3
Vertical jump (cm), Mean ± SD	30.63 ± 7.2	24.10 ± 8.3
Working memory, Mean ± SD	6.35 ± 1.5	5.91 ± 1.1
VO_2_ max (ml/kg/min), Mean ± SD	44.70 ± 9.9	34.03 ± 7.9
DNAmPhenoAge, Mean ± SD	46.96 ± 9.3	51.31 ± 12.0
AgeAccelPheno, Mean	− 0.43	0.63
DNAmGrimAge, Mean ± SD	58.53 ± 8.5	62.09 ± 9.9
AgeAccelGrim, Mean	− 0.25	0.33

### Physiological tests

Body mass and height were measured and the body mass index was described by the body composition monitor BF214 (Omron, Japan). Relative maximum hand gripping force a measure of age-associated decline in general muscle strength (Eika et al. 2019) was assessed by the CAMRY EH101 dynamometer. Relative maximal oxygen uptake is one of the best markers of viability and a higher level of VO2max is associated with decreased levels of a wide range of diseases (Hawkins and Wiswell 2003; Carnethon et al. 2005). We used the Chester step test to appraise the level of VO2max (Izquierdo et al. 2019). A Digit span test was applied to assess the working memory (Martinez-Diaz et al. 2020), where larger values indicate better verbal short-term memory.

### Determination of hematologic and telomere length

Determination of hematologic and biochemical variables, blood samples were collected before the subjects performed the VO2max evaluation test, and were stored in evacuated tubes containing EDTA as an anticoagulant. Blood samples were centrifuged and stored at − 80 °C degrees. The biochemical tests were carried out in the Clinical Analysis Laboratory of Semmelweis University, Budapest.

#### DNA isolation

DNA was isolated from the K2-EDTA anticoagulated blood samples using a DNA isolation kit (Pure LinkTM Genomic DNA Mini kit, Thermo Fisher, Carlsbad, CA, USA), according to the manufacturer’s instructions.

#### Measurement of telomere length

The average relative telomere length of genomic DNA was determined from whole blood samples using Cawthon’s PCR-based method (Wan et al. 2019) with the commercially available PCR kit (ScienCell Research Laboratories inc., San Diego CA Catalog no. #8908). During this reaction, telomere-specific primers recognize and amplify telomeric sequences. For each DNA sample, two consecutive reactions were performed: the first for amplification of a single-copy reference (SCR) gene and the second for the telomeric sequence. The former recognizes and amplifies a 100 bp region on human chromosome 17 and serves as a reference for calculating the telomere length of the target samples. The PCR reactions were performed in a final volume of 20 μl. We used 5 ng reference/genomic DNA sample (final concentration = 0.625 ng/μl), 2 μl telomere primer, and 10 μl 2XMaster Mix, PCR conditions were as follows: first 95 °C for 10 min, followed by 32 cycles of 95 °C for the 20 s, 52 °C for 20 s, and 72 °C for 45 s. All samples were tested in triplicate.

In addition, the telomere length was also evaluated by Horvath’s software which estimates the telomere length from methylation (Pearce et al. 2022).

#### Measurement of DNA methylation

Epigenome-wide DNA methylation 850 K was measured with the Infinium MethylationEPIC BeadChip (Illumina Inc., San Diego, CA) according to the manufacturer’s protocol. Briefly, 500 ng of genomic DNA was bisulfite converted using the EZ-96 DNA Methylation MagPrep Kit (Zymo Research, Irvine, CA, USA) with the KingFisher Flex robot (Thermo Fisher Scientific, Breda, Netherlands). The samples were plated in randomized order. The bisulfite conversion was performed according to the manufacturer’s protocol with the following modifications: For binding of the DNA 15 µl MagBinding Beads were used. The conversion reagent incubation was done according to the following cycle protocol: 16 cycles of 95 °C for 30 s followed by 50 °C for 1 h. After the cycle protocol, the DNA was incubated for ten minutes at 4 °C. Next, DNA samples were hybridized on the Infinium MethylationEPIC BeadChip (Illumina Inc., San Diego, CA) according to the manufacturer’s protocol with the modification that 8 µl bisulfite-treated DNA was used as starting material. Quality Control of the DNA methylation data was performed using, Meffil and Ewastools packages with R version 4.0.0 (Min et al. 2018, Murat et al. 2020).

Acceleration for PhenoAge and GrimAge is defined as the raw residuals i.e. the difference between the observed and the expected value when the methylation-based age estimator regressed on chronological age. Indeed, methylation-based clocks are better at predicting health outcomes if blood cell composition is incorporated (Chen et al. 2016). Both PhenoAge and GrimAge are known to be associated with changes in leukocyte distribution like the decrease in naive CD8 cells, increase in granulocytes, etc. However, after blood cell count adjustment the decrease in p-value is minimal for GrimAgeAccel (Lu et al. 2019) as well as for PhenoAgeAccel (Levine et al. 2018).

Samples that failed technical controls, including extension, hybridization, and bisulfite conversion, according to the criteria set by Illumina, were excluded. Samples with a call rate < 96% or at least 4% of undetected probes were also excluded. Probes with a detection p-value > 0.01 in at least 10% of the samples were set as undetected. Probes with a bead number < 3 in at least 10% of the samples were excluded. We used the "noob" normalization method in R to quantify the methylation level (Triche et al. 2013). The details on the processing of DNAm data and the calculation of the measures of aging, or pace of aging, were calculated using Horvath’s online age calculator (https://dnamage.genetics.ucla.edu/).

#### Statistics

The results were subjected to statistical tests. Statistical analysis was done by using Statistica 13 software (TIBCO). After testing for normal distribution, the relevant parametric and non-parametric test methods were applied. The differences between groups were examined by multiway ANOVA. The interdependence of the individual variables was analyzed using multivariate regression analysis.

## Results

**Fig. 1:**
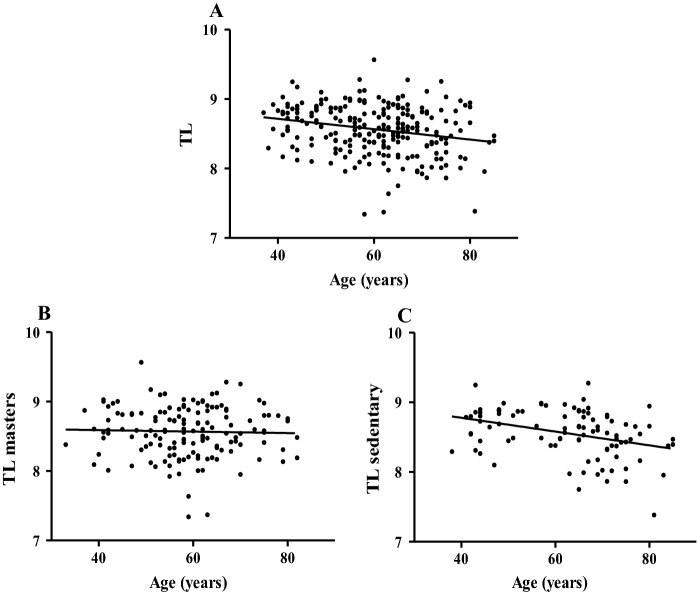
The association between telomere length and chronological age. Telomere length measured by RT-PCR negatively correlated with chronological age in all cases (Panel **A** N = 241) and in sedentary (Panel **C** N = 95), but not in masters (Panel **B** N = 146)

We have measured or estimated the TL with two different methods one based on RT-PCR measurements while the other was calculated methylation-based estimation of TL. There were no significant differences between the telomere length of masters and sedentary, measured or estimated (TL: masters mean 8.57 ± 0.36, sedentary mean 8.56 ± 0.34; DNAmTL masters mean 6.85 ± 0.27, sedentary mean 6.79 ± 0.29). The results from the RT-PCR measurements revealed that the TL showed a weak, but significant negative relationship with the chronological aging of the subjects (r =  − 0.23; p = 0.0003) (Fig. 1A). However, we could not detect a significant correlation between age and TL in masters (masters: r =  − 0.03; p = 0.7142 vs sedentary: r =  − 0.35; p = 0.0004) (Fig. 1B, C).

**Fig. 2:**
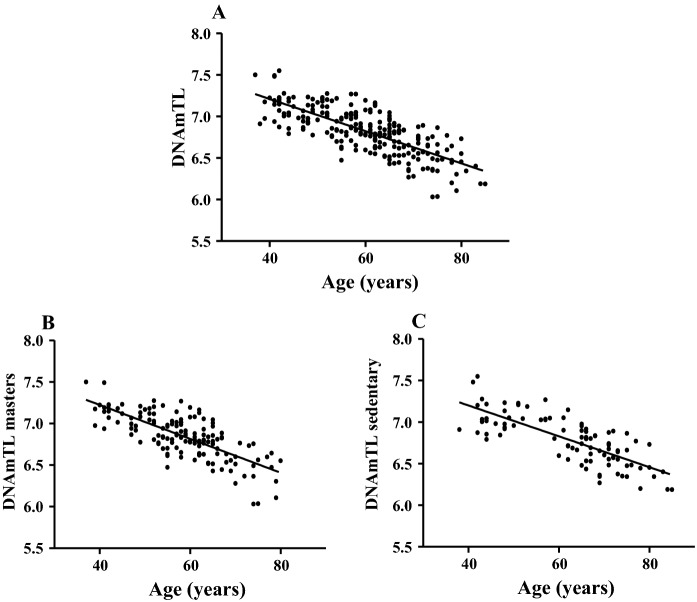
The association between estimated telomere length and chronological age. Estimated telomere length negatively correlated with chronological age in all cases (Panel **A** N = 241) in the masters (Panel **B** N = 146) and in sedentary (Panel **C** N = 95)

As far as the DNAm-based estimation was concerned significant relationships were found in the whole cohort, both masters and controls (Fig. 2A–C). The evaluation of the relationship between DNAmTL and TL revealed a significant relationship (r = 0.366, p < 0.0001).

**Fig. 3:**
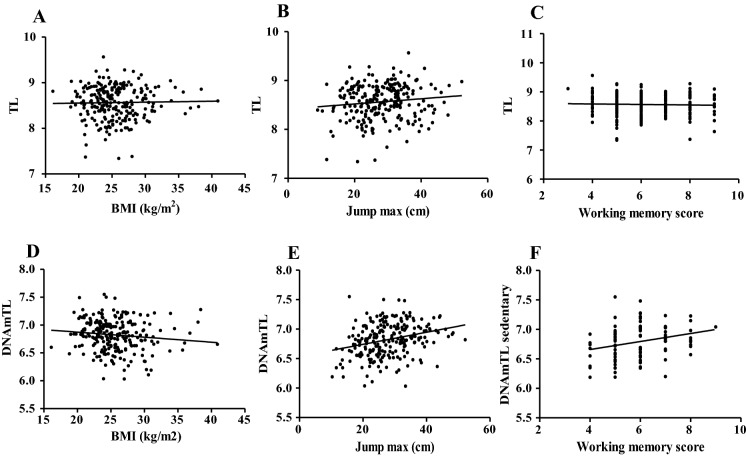
The relationship between physiological test results and telomere length. A significant relationship was not present between RT-PCR-based telomere length, body mass index (BMI, Panel **A**), maximal vertical jump height (Jump max, Panel **B**), and working memory (Working memory score, Panel **D**) N = 241. Panel **E**, (N = 241), **F** (N = 95) show DNAm-based results

According to results measured by RT-PCR, there is no relationship between BMI, (r =  − 0.02; p = 0.7450, Fig. 3A; masters: r =  − 0.0356; p = 0.6698, sedentary: r = 0.099; p = 0.3419), vertical jump results (r = 0.12; p = 0.0689, Fig. 3B; masters: r = 0.15; p = 0.0705, sedentary: r = 0.086; p = 0.4097), and the scores of working memory (r =  − 0.032; p = 0.622, Fig. 3C; masters: r =  − 0.097; p = 0.2450, sedentary: r =  − 0.097; p = 0.2450). Despite this, the DNAm-based estimation of TL showed similar results with BMI (r =  − 0.12; p = 0.0668, Fig. 3D; masters: r =  − 0.09; p = 0.2869, sedentary: r =  − 0.096; p = 0.3595), the vertical jump results correlated significantly with DNAmTL (r = 0.29; p < 0.0001, Fig. 3E; masters: r = 0.21; p = 0.0106, sedentary: r = 0.336; p = 0.0010). Working memory also showed a positive correlation with DNAmTL, but only in controls (r = 0.085; p = 0.1956, Fig. 3F; masters: r =  − 0.042; p = 0.6193, sedentary: r = 0.264; p = 0.0104).

**Fig. 4:**
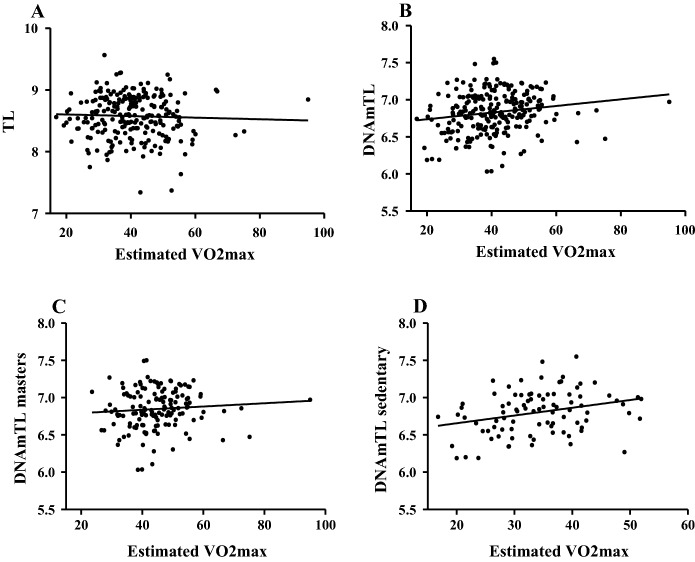
The relationship between cardiovascular fitness (VO2max) and telomere length. The maximal oxygen uptake was estimated from the step test results and a significant relationship was neither found in the total number of subjects when assessed by RT-PCR gained results (Panel **A** N = 241). DNAm-based TL calculation, on the other hand, showed significant relationships in all cases (Panel **B** N = 241) and in sedentary (Panel **D** N = 95), but not in masters (Panel **C** N = 146)

The maximal oxygen uptake (VO2max) which was calculated from the Chester step test, was higher in athletes (masters 44.7 ml/kg/min vs. sedentary: 33.7 ml/kg/min). The results showed no relationship with RT-PCR-based TL (r =  − 0.039; p = 0.5484 Fig. 4A; masters: r =  − 0.1; p = 0.2286, sedentary: r = 0.12; p = 0.2488). On the other hand, the DNAm-based TL estimate showed a significant correlation with VO2max in all subject cases (r = 0.17; p = 0.0094, Fig. 4B) and in sedentary (r = 0.29; p = 0.0067, Fig. 4C), but not in masters (r = 0.08; p = 0.3414, Fig. 4D).

Since telomere length is often to be associated with morbidity (Cheng et al. 2021) and lifestyle-related factors like tobacco smoking (Astuti et al. 2017), we made a supplementary analysis (multiple regression) with suspected confounders variables to check for possible biases. In the multivariable models, boolean variables for gender, smoking, high blood pressure, autoimmune disease, asthma, diabetes, and cardiovascular disease were included. After adding the mentioned variables into the model only DNAmTL showed a significant correlation with exercise physiology variables (VO2max: partial r = 0.267, p = 4.8E-6, JumpMax: partial r = 0.44, p = 2.89E-12).

**Fig. 5:**
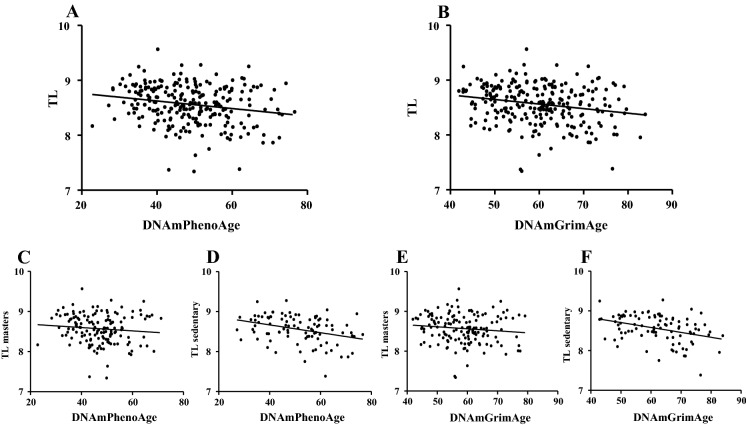
The correlation between telomere length and DNAmPhenoAge and DNAmGrimAge measured by RT-PCR. The epigenetic aging was calculated on the DNA methylation pattern based on the description of DNAmPhenoAge and DNAmGrimAge. Both the DNAmPhenoAge (Panel **A** N = 241) and DNAmGrimAge (Panel **B** N = 241) showed a significant relationship with RT-PCR-based TL. Exercise abolished these correlations (Panel **C**–**F** N = 241)

When the possible association between RT-PCR measurement-originated data and DNA methylation-based epigenetic aging was examined, it turned out that in all subjects, a case both DNAmPhenoAge and DNAmGrimAge related to TL (r =  − 0.21; p = 0.0012; and r =  − 0.22; p = 0.0007) respectively (Fig. 5A, B). However, this connection seems to be disappearing due to long-term training (DNAmPhenoAge; masters: r =  − 0.11 p = 0.2014, sedentary: r =  − 0.34; p = 0.0007, Fig. 5C, D) (DNAmGrimAge masters: − 0.12 p = 0.1574, sedentary: r =  − 0.36; p = 0.0004, Fig. 5E, F).

**Fig. 6:**
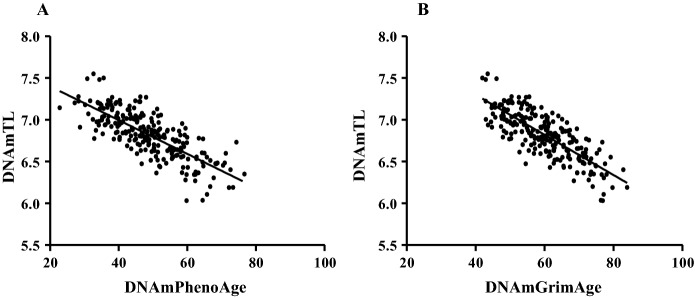
The relationship between DNAm-based TL estimation with DNAmPhenoAge and DNAmGrimAge. DNAm-assessed TL showed an even stronger relationship with DNAmPhenoAge and DNAmGrimAge (Panel **A**, **B**). N = 241

The relationship between DNAm-based TL estimation with DNAmPhenoAge and DNAmGrimAge showed an even more powerful relationship (r =  − 0.77; p < 0.0001; and r =  − 0.79; p < 0.0001, Fig. 6A, B), but exercise habits did not affect this (Table 2).

**Table 2:** Correlation between DNA methylation-based markers

DNAmTL	All	Masters	Sedentary
DNAmPhenoAge	r = − 0.77; p < 0.0001	r = − 0.75; p < 0.0001	r = − 0.79; p < 0.0001
PhenoAgeAccel	r = − 0.21; p = 0.0012	r = − 0.16; p = 0.062	r = − 0.26; p = 0.0126
DNAmGrimAge	r = − 0.79; p < 0.0001	r = − 0.76; p < 0.0001	r = − 0.82; p < 0.0001
GrimAgeAccel	r = − 0.25; p < 0.0001	r = − 0.28; p = 0.0006	r = − 0.20; p = 0.0497

When the relationship of DNAmPhenoAge acceleration (r =  − 0.21; p = 0.0012) and DNAmGrimAge acceleration (r =  − 0.25; p < 0.0001) with DNAm based TL estimation were evaluated, the results revealed that longer telomeres were related to decelerated aging except for DNAmPhenoAge acceleration (Table 2). However, this at least a part could be due to some possible overlapping of CpGs incorporated in DNAnPhenoAge/GrimAge and DNAmTL.

## Discussion

In the present study, our data revealed that TL has associated with DNA methylation-based epigenetic aging biomarkers. It has been known that a higher level of cardiovascular fitness, the VO2max is associated with longer TL in a wide range of age groups (18–72 years old) (LaRocca et al. 2010). A recent systematic review screened the results of 43 randomized controlled, observational or interventional studies (Schellnegger et al. 2022) and concluded positive effects of exercise on telomere dynamics, however, the contribution of training modalities (intensity, duration, type of exercise, etc.) of the beneficial effects are not known.

It is clear that one of the striking effects of aging is a suppressed physiological function, however, it is also known that the progress of aging depends on environmental and lifestyle factors, including physical fitness (Radak et al. 2019). Indeed, the DNA methylation-based epigenetic biomarkers reflect individual aging more precisely than chronological aging and are related to telomere length (Lee et al. 2019). Based on our results, it cannot be excluded that exercise-induced DNA methylation is associated with longer telomere. However, it has to be noted that since the present study is a cross-sectional study, the possible relationship between the level of physical fitness a TL must consider with caution. Moreover, both DNAmPhenoAge acceleration and DNAmGrimAge acceleration showed that a higher level of physical fitness suppresses the progress of aging. The health-promoting effects of exercise are well documented (Hortobágyi et al. 2022; Quan et al. 2020; Radak et al. 2013), and this study also demonstrates that these changes could be associated with DNA methylation.

Surralles et al. (1999) demonstrated that telomere shortening could be affected by histone acetylation, therefore influenced by epigenetics. Moreover, the same study found that longer-lived cell lineages have an active X chromosome with a longer telomere than the inactive X, indicating that telomere maintenance alleles on the X chromosome impact survival. It was also suggested that the male Y chromosome is less protected and more prone to telomere shortening, but the chromosome-dependent telomere shortening is under debate (Genovesi et al. 2021). The results of the current study could not demonstrate that in the given population females have longer telomeres than males. It was suggested that there is a gender difference in the length of telomere, based on the telomerase activity, which could add base pairs to telomere is influenced by estrogen (Kyo et al. 1999), however, the study of Lin et al. reported that postmenopausal women who had longer endogenous estrogen therapy had longer telomere length with lower telomerase activity (Lin et al. 2011). Therefore, the mechanism of telomere shortening with gender bias appears to be complex and requires further investigations (Barrett and Richardson 2011).

The associations between TL and physiological functions are very important (Nordfjäll et al. 2008; Colon et al. 2019; Buttet et al. 2022). There are a few reports on TL and VO2max (LaRocca et al. 2010; Østhus et al. 2012; Brandao et al. 2020; Werner et al. 2019). Cross-sectional and longitudinal studies suggest that endurance exercise health-promoting effects are associated with longer TL. The underlying mechanisms are not known, but they could link to exercise-induced upregulation of antioxidant systems (Radak et al. 2013) since increased oxidative stress is associated with shorter telomeres (D'Mello et al. 2015).

The age-related loss of cognitive and physical performance is very normal, but the degree of loss and values are very much related to the level of physical fitness (Booth and Roberts 2008). Genetics can influence trainability, but regular exercise can greatly improve the level of physical fitness (Radak and Taylor 2022). In the present study, we have shown that DNAm-based estimation is a more sensitive method to examine the relationship between TL and physiological function, especially with VO2max than the RT-PCR-based method. In conclusion, the results of this study further emphasize the importance of the level of physical fitness in the aging process.
